# Supplementary material for: Hippocampal Resting State Functional Connectivity Associated with Physical Activity in Periadolescent Children
Source: Brain Sci. 2023 Nov 7;13(11):1558. doi: 10.3390/brainsci13111558 (PMC10669534; doi:10.3390/brainsci13111558)
Supplement: Supplementary file 1 [file brainsci-13-01558-s001.zip › brainsci-2653705-supplementary.pdf]

Supplementary Materials

Supplementary Figure S1. Lateral, medial, and dorsal brain views of hippocampal rs-FC

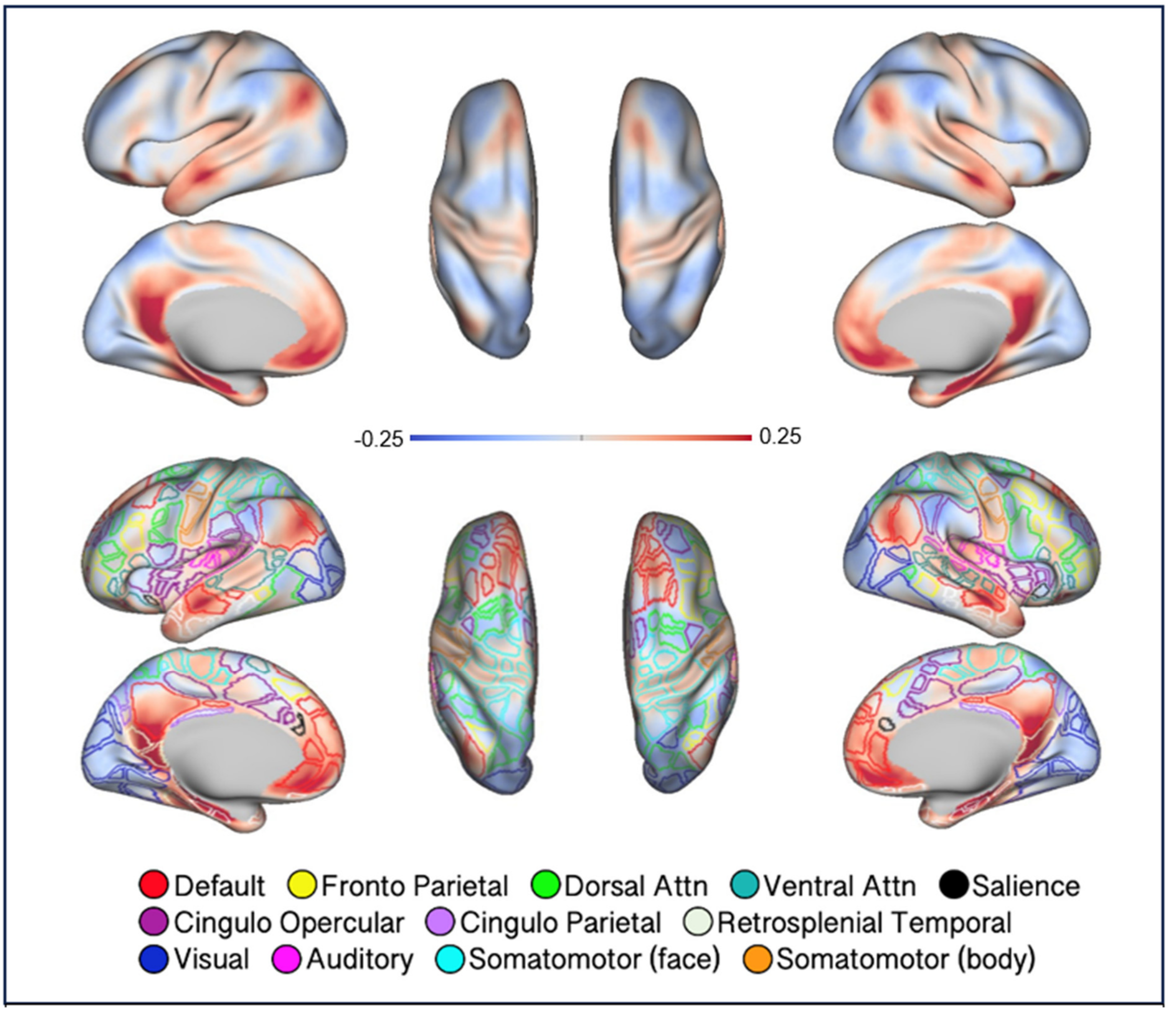

Supplemental Figure S1. (Top) Lateral, medial, and dorsal brain views of mean hippocampal rs-FC, and (bottom) lateral, medial, and dorsal brain views of mean hippocampal rs-FC overlayed with Gordon's 333 parcellation.

Supplementary Figure S2. Lateral, medial, and dorsal brain views of rs-FC covarying with physical activity

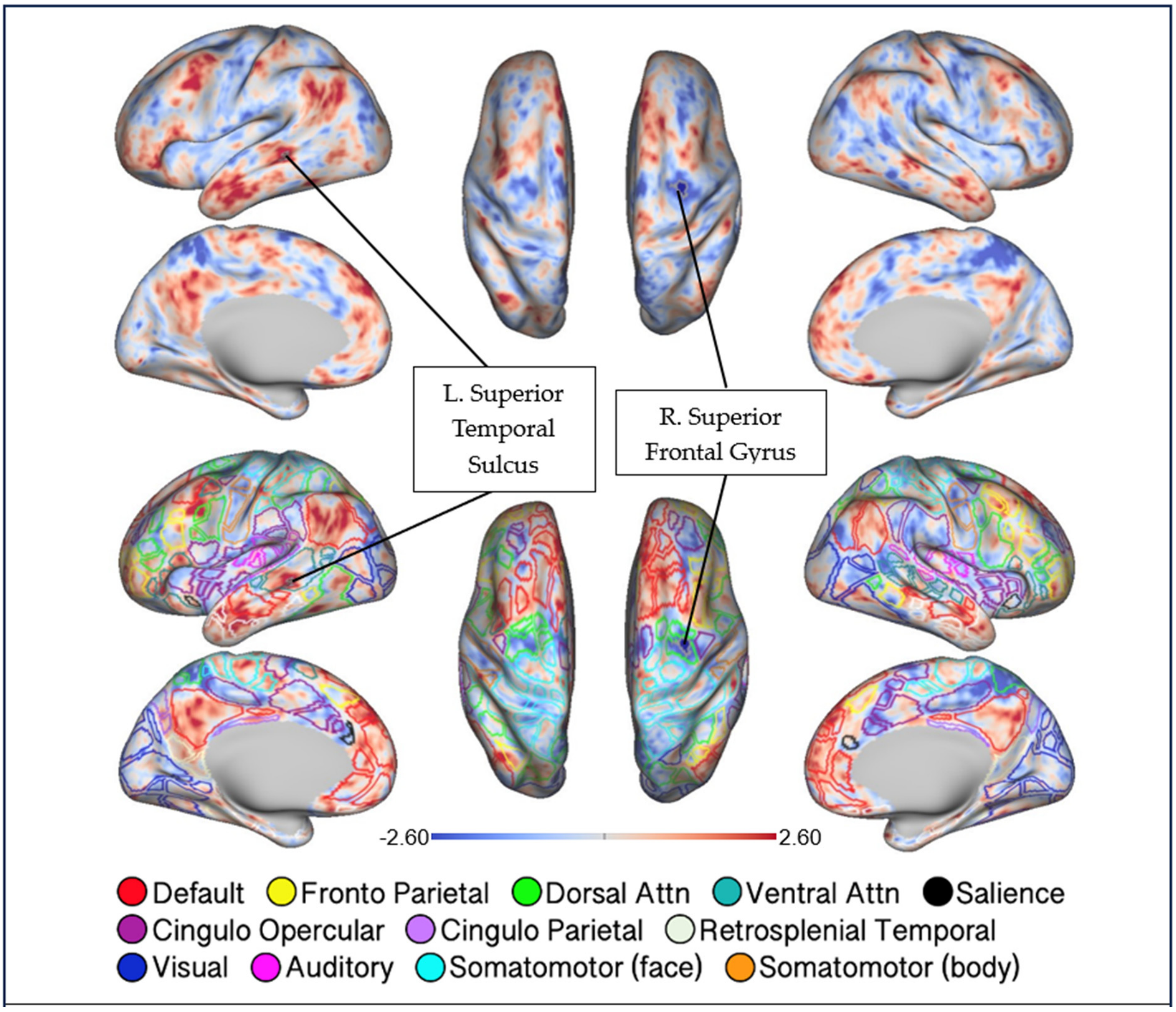

Supplemental Figure S2. (Top) Lateral, medial, and dorsal brain views of rs-FC covariation with self- reported physical activity, with significant clusters outlined in white, and (bottom) overlayed with Gordon's 333 parcellation.
